# Diversity, Ecological Role and Biotechnological Potential of Antarctic Marine Fungi

**DOI:** 10.3390/jof7050391

**Published:** 2021-05-17

**Authors:** Stefano Varrella, Giulio Barone, Michael Tangherlini, Eugenio Rastelli, Antonio Dell’Anno, Cinzia Corinaldesi

**Affiliations:** 1 Department of Materials, Environmental Sciences and Urban Planning, Polytechnic University of Marche, Via Brecce Bianche, 60131 Ancona, Italy; 2 Institute for Biological Resources and Marine Biotechnologies, National Research Council (IRBIM-CNR), Largo Fiera della Pesca, 60125 Ancona, Italy; giulio.barone@irbim.cnr.it; 3 Department of Research Infrastructures for Marine Biological Resources, Stazione Zoologica “Anton Dohrn”, Fano Marine Centre, Viale Adriatico 1-N, 61032 Fano, Italy; michael.tangherlini@szn.it; 4 Department of Marine Biotechnology, Stazione Zoologica “Anton Dohrn”, Fano Marine Centre, Viale Adriatico 1-N, 61032 Fano, Italy; eugenio.rastelli@szn.it; 5 Department of Life and Environmental Sciences, Polytechnic University of Marche, Via Brecce Bianche, 60131 Ancona, Italy; a.dellanno@univpm.it

**Keywords:** marine fungi, mycology, fungal diversity, Antarctica, bioprospecting, psychrophiles, cold-adapted enzymes, industrial applications, blue biotechnologies

## Abstract

The Antarctic Ocean is one of the most remote and inaccessible environments on our planet and hosts potentially high biodiversity, being largely unexplored and undescribed. Fungi have key functions and unique physiological and morphological adaptations even in extreme conditions, from shallow habitats to deep-sea sediments. Here, we summarized information on diversity, the ecological role, and biotechnological potential of marine fungi in the coldest biome on Earth. This review also discloses the importance of boosting research on Antarctic fungi as hidden treasures of biodiversity and bioactive molecules to better understand their role in marine ecosystem functioning and their applications in different biotechnological fields.

## 1. Introduction

The Antarctic ecosystem is one of the most hostile environments on Earth [1]. Despite the harsh environmental conditions (extremely low temperatures, prolonged periods of darkness, and high levels of ultraviolet radiations), Antarctica hosts a variety of unique organisms, from penguins and other endemic birds to whales, seals, fish, and invertebrates inhabiting both the land and the Southern Ocean [2]. Although the number of species inhabiting the Antarctic mainland is low compared to other terrestrial environments [3], the marine ecosystems host an unexpected high biodiversity and an ever-increasing number of species is being reported every year [4,5,6,7]. For example, the number of invertebrate marine Antarctic species has been estimated to range from 17,000 to 20,000, only 8000 of which have been described to date [6,8]. However, these estimates are likely underrating the overall Antarctic marine diversity due to the low sampling effort and limited spatial coverage of the studies conducted [5,7,9]. Moreover, molecular techniques are now enabling the identification of cryptic and previously unknown species, thus boosting our current ability in assessing Antarctic biodiversity [10].

Many factors can promote the high biodiversity in the Southern Ocean, including high environmental heterogeneity, isolation, and low human impact [4]. Indeed, coastal habitats in Antarctica are characterized by a wide spatial heterogeneity caused by high variability in nutrient dynamics, light availability, and extensive seascape variations due to ice formation and melting which determine major changes in thermohaline conditions, biological productivity, and sedimentation processes. Moreover, Antarctica’s geographic and oceanographic isolation has allowed many new species to evolve in the absence of competition from lower latitudes’ vicariants [4]. Overall, these factors have contributed to shape Antarctic biodiversity in a unique way [11].

The diversity of large organisms inhabiting Antarctic ecosystems has received a larger attention than the diversity of microbial assemblages, although the microbial component represents an important fraction of the whole biomass and plays pivotal roles in biogeochemical cycles and marine food web functioning [12,13,14,15,16]. There is also evidence that microbial diversity represents a major reservoir of novel taxa, biochemical pathways, genes and compounds with biotechnological applications [17,18,19,20].

Despite the harsh conditions, fungi are ubiquitously present in Antarctic ecosystems [21]. This success is largely due to the vast array of fungal enzymes, secondary metabolites, and bioactive molecules, which allowed fungal colonization and diversification in almost every habitat on Earth [22]. Their peculiar biological adaptions to low temperatures lead to the production of structurally novel enzymes and bioactive metabolites, which provide fungi competitive advantages over other microorganisms through chemically-mediated interactions, defense, and virulence factors for plants and animals [23,24,25,26]. In the last decade, several studies, exploring the fungal diversity in Antarctic marine environments, have revealed promising properties of fungi relevant for biotechnological applications (e.g., pharmaceutical, food, and cosmeceutical industries [27,28,29]). Indeed, increasing evidence indicates that cold-adapted fungi are a relevant target to the present and future scientific research for their possible biotechnological applications, including the development of new drugs and exploitation in several industrial processes [30,31].

In this review we collected the current literature about fungi in Antarctic marine ecosystems, focusing on their taxonomic diversity and ecological functions, as well as their potential for developing new blue biotechnologies.

## 2. Fungal Diversity and Ecology in Antarctic Marine Environments

**Figure 1:**
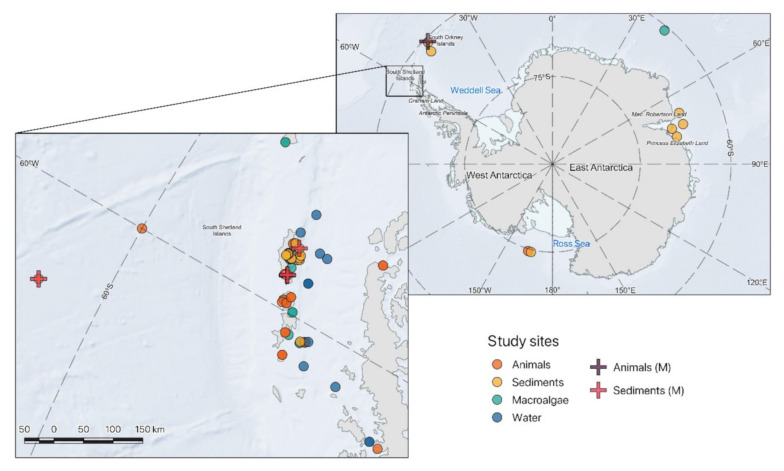
Locations of the fungi identified from different Antarctic marine substrates: animals (orange circle), sediments (yellow circle), macroalgae (green circle), and water (blue circle) based on culture dependent approaches or identified through metagenomic analysis: animals (purple cross) and sediments (pink cross) (for detailed elucidation on the samples where fungal taxa were isolated, and coordinates see Table S1).

Fungi are widely distributed in marine Antarctic ecosystem, and their occurrence has been recorded in seawater and sediments as well as associated with macroalgae and invertebrates [27]. Marine fungi are supposed to contribute to population dynamics, C and nutrient cycles in the oceans [32], and yet only a limited number of studies have investigated fungal diversity and ecology of Antarctic marine fungi. These studies mainly focused on the identification of fungi and yeasts isolated from waters, sediments, animals, and/or macroalgae (Figure 1). So far, most of the information has been acquired through culture-dependent approaches on samples collected from the Shetland Islands, leaving most coastal and offshore habitats unexplored.

### 2.1. Fungal Diversity

#### 2.1.1. Fungi in Antarctic Marine Environment

**Figure 2:**
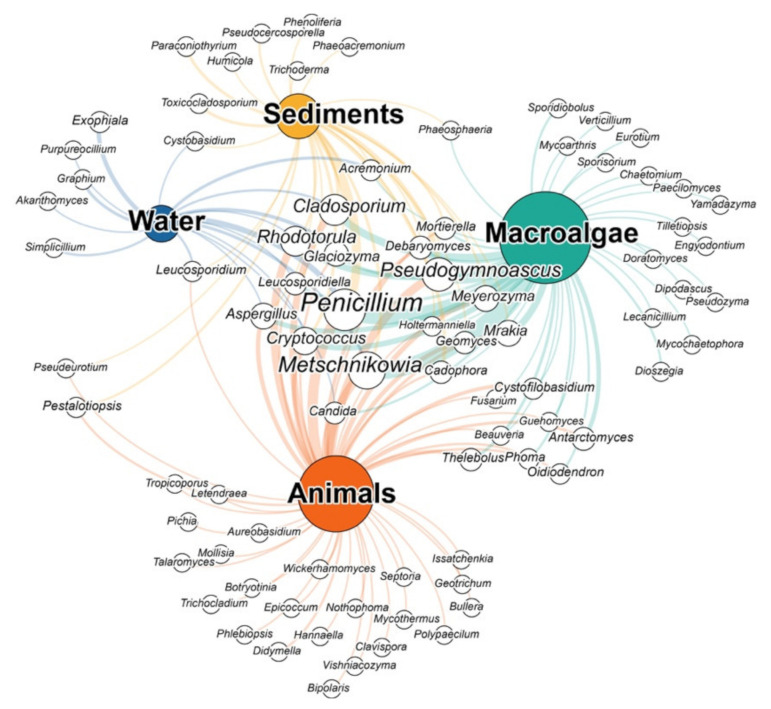
Network diagram displaying the records of Antarctic fungal taxa belonging to different genera identified through culture dependent approaches or metagenomic analyses in the four most commonly marine matrices (water, sediments, animals, macroalgae). The size of the white nodes is proportional to the number of records in the studies in which the genus has been found, while the size of coloured nodes is proportional to the overall number of genera retrieved.

In the last years, culture-dependent and molecular techniques have allowed us to describe a large number of fungal taxa in seawater [33,34]. Likewise, fungi have been identified in several polar environments [35,36,37]. To date, only a few studies have successfully identified filamentous fungi and yeasts in both coastal and offshore Antarctic waters [38,39]. These studies, employing culture-dependent methods, have been able to isolate a number of fungal genera belonging to the orders *Eurotiales*, *Hypocreales*, *Chaetothyriales,* and *Kriegeriales*. In particular, Antarctic waters seem to exclusively host the genera *Exophiala*, *Graphium*, *Simplicillium, Purpureocillium,* and *Akanthomyces*. These genera are known to include parasites, pathogens, and likely saprotrophs, which may be involved in complex interactions within the water column [40,41,42]. However, other genera found in Antarctic waters such as *Penicillium*, *Metschnikowia*, *Rhodotorula*, and *Glaciozyma* have also been found elsewhere in Antarctica (Figure 2; Table S2). Within the water column, fungi can have significant effects on primary production dynamics and carbon fluxes within the marine food webs, by acting as saprotrophs and interacting with marine phytoplankton [37,43,44].

Despite the increasing number of investigations carried out worldwide, to date, fungal diversity in Antarctic sediments has been explored in a relatively limited number of studies, which mainly focused on the diversity of culturable fungal species [45,46,47,48,49,50,51,52,53,54,55,56,57,58,59,60,61]. Recently, for the first time, molecular tools have been employed to investigate the fungal diversity in Antarctic marine sediments and allowed the identification of a large number of fungal taxa although much of the fungal diversity in Antarctic marine sediments still remains unknown [49].

Antarctic marine sediments have been shown to host a plethora of fungal taxa. For example, the genera *Pseudocercosporella, Toxicocladosporium, Trichoderma, Humicola, Paraconiothyrium, Phaeoacremonium,* and *Phenoliferia* have been documented exclusively in marine sediments and to be potentially involved in the degradation of organic matter. Nonetheless, fungal diversity in marine sediments also include many other genera found in other Antarctic habitats, such as *Penicillium*, *Metschnikowia*, *Rhodotorula,* *Cladosporium* and *Glaciozyma* (Figure 2). The genus *Pseudogymnoascus* genus found in Antarctic sediments has been also recorded in other cold environments including polar regions and glaciers [27,49,62,63], and the genus *Phaeosphaeria*, whose members include plant pathogens, has been also found in association with the Antarctic macroalgae *Adenocystis utricularis* [64,65].

#### 2.1.2. Fungi Associated with Antarctic Macroalgae and Animals

Most of the research on Antarctic fungi focused on the assemblages associated with benthic macroalgae of the South Shetland Islands (Antarctic Peninsula) [54,64,66,67,68,69,70]. In particular, the macroalgae *Adenocystis utricularis*, *Desmarestia anceps* and *Palmaria decipiens* host a rich fungal diversity, including genera like *Pseudogymnoascus*, *Antarctomyces*, *Oidiodendron*, *Penicillium*, *Phaeosphaeria*, *Cryptococcus*, *Leucosporidium*, *Metschnikowia*, and *Rhodotorula*. Some of the species belonging to these genera appear to be endemic of Antarctica (i.e., *Antarctomyces pellizariae*, *Antarctomyces psychrotrophicus, Cryptococcus victoriae*, *Cryptococcus adeliensis*, *Metschnikowia australis, Pseudogymnoascus pannorum, Mortierella antarctica*), while others appear rather ubiquitously distributed (i.e., *Penicillium* sp., *Aureobasidium* sp. and *Rhodotorula* sp.) [64,66,68,69]. Despite this, fungi associated with macroalgae are apparently unique since several genera have never been found in any other marine sample (Figure 2). This rich and diverse fungal assemblages can have an important ecological role since they may produce enzymes with the potential to degrade algal detritus and may be involved in organic matter cycling and energy transfer within the marine food web [69].

Analogously to what has been reported for macroalgae, fungi have been isolated also from marine animals, with which they can interact as pathogens, parasites, or symbionts [71,72,73,74,75,76,77,78]. Nevertheless, the nature and the strength of the relationships between the host and the associated fungi have yet to be fully understood [78]. Fungal diversity has been investigated in a variety of Antarctic organisms such as sponges, annelids, crustaceans, molluscs, and echinoderms collected in the South Shetland Islands (Figure 1), mainly through culture-dependent approaches [54,79,80,81,82,83]. Although culture-based studies allowed isolating and investigating a variety of fungal taxa, molecular tools have allowed the identification of a larger fraction of the fungal diversity provided. In particular, such molecular methods provided new information about the fungal diversity associated with the Antarctic Krill, *Euphausia superba* [84], and the marine sponges *Leucetta antarctica* and *Myxilla* sp. [85]. Several other fungal taxa, including members of the orders Saccharomycetales and Eurotiales, and of the families *Saccharomycetaceae* and *Aspergillaceae,* have been identified. In particular, the most represented taxa included ubiquitous genera such as *Rhodotorula*, *Penicillium, Metschnikowia*, *Aspergillus*, as well as 23 different exclusive genera such as *Wickerhamomyces*, *Geotrichum, Letendraea*, and *Bullera* (Figure 2). Among them, *Wickerhamomyces* spp. has particular characteristics: It can in fact grow under extreme environmental stressful conditions, such as low and high pH, high osmotic pressure, absence of oxygen, and also shows antimycotic activity [86]. Despite further studies being needed to better understand the ecological significance of fungal–host interactions, there is evidence that these fungi can be involved in defensive mechanisms by producing cytotoxic, antimycotic, and antibiotic molecules, which could increase the animal’s wellbeing [83]. For instance, fungi associated with the Antarctic krill can be involved in the defense mechanisms of the host against pathogenic bacteria [83]. Nevertheless, a number of fungal pathogens (i.e., *Rhodotorula*, *Debaryomyces*) have been isolated from marine organisms which have the ability to compromise host health and fitness, but their possible detrimental effects on marine fauna have not yet been estimated.

### 2.2. Contribution of Fungi to Ecological Processes in Antarctic Marine Ecosystems

While on terrestrial ecosystems the role of fungi is largely recognized, their ecological role in Antarctic marine ecosystems has yet to be understood. In the last decade, several studies highlighted that fungi are widely distributed in marine environments, from coastal waters [87] to the deep-sea surface and subsurface sediments [88,89,90,91,92,93,94], extreme habitats such as hypersaline anoxic basins [95,96,97], cold seeps [98,99,100], hydrothermal vents [101,102], and oil reservoirs [103]. Polar environments are also characterized by a considerably high number of fungal taxa [36,55,104,105,106], which can be involved in organic matter degradation and nutrient cycling, as well as in intimate relationships with a variety of organisms [27,32,49,107].

In Antarctic marine waters, owing to their unique enzymatic capabilities and metabolic versatility [29,108,109], fungi can be important components for the biogeochemical processes and the functioning of the food webs [110]. As saprotrophs, some fungi may utilize phytoplankton-derived detritus contributing to organic matter fluxes [111], while others can interact with planktonic microorganisms influencing ecological dynamics and food webs [112]. The same Authors have provided a theoretical framework to describe how aquatic fungi interact with their environment, introducing the concept “mycoflux” to refer to the interactions between pelagic fungi and other microbes and their effects on the carbon pump [112]. Previously, Kagami et al. (2014) introduced the concept “mycoloop” to indicate the parasitic interaction between fungi and other planktonic components, suggesting that parasitic fungi can facilitate energy transfer from phytoplankton to zooplankton [113]. This concept is also supported by recent metabarcoding and metagenomic studies carried out in different aquatic environments [37,114,115], which revealed a high diversity of parasitic (or facultative parasitic) zoosporic fungi associated with phytoplankton and zooplankton [43,113,116,117,118,119,120]. Overall, these findings suggest that fungal parasites can be important in influencing the aquatic food webs as other planktonic parasites [121].

Even in Antarctic benthic ecosystems, fungi can have an important role in C cycling and nutrient regeneration processes. Benthic fungi, acting as decomposers of organic matter can be involved in the degradation of recalcitrant organic compounds, which otherwise accumulate in marine sediments [93,122,123], and may mediate C and energy transfer to higher trophic levels [112,124,125,126]. The association of fungi with microalgal [43] and macroalgal detritus can improve the nutritional value of organic matter by lowering the carbon:nitrogen:phosphorus ratio [127,128]. Moreover, fungi in Antarctic benthic ecosystems are likely involved in ecological interactions with other eukaryotes, similarly to what observed in other extreme marine environments acting as pathogens and parasites [129,130].

In Antarctic benthic coastal ecosystems, an important relationship between fungi and macroalgae has been reported [64]. Macroalgae are ecosystem engineers that contribute to primary production in cold and temperate coastal marine environments [131]. Macroalgae represent the second biggest reservoir of fungal diversity after sponges [132], and the relationship between the host and its fungal assemblage encompasses mutualism, parasitism, and saprophytism [133,134,135]. In this relationship, fungi can provide important advantages for the growth, defence, development, and nutrition of the algal host [136,137,138]. However, several fungi can also act as pathogens, compromising the host’s health and its ecological functions [139].

Fungi have also been documented in association with Antarctic benthic marine animals [27,54], but so far, the nature and the mechanisms of these relationships remain mostly unknown. To date, only one study based on a functional analysis of fungi associated with sponges highlighted that fungi can have an important role in the degradation of the organic matter, contributing to nutrient cycling and in turn influencing the carbon fixation pathways of prokaryotes and other micro-eukaryotes within the microbial assemblages [85].

Paradoxically, the biotechnological focus of the Antarctic marine fungi has contributed to accumulate more information on their potential industrial applications than on their quantitative relevance and role in biodiversity and functioning of Antarctic marine ecosystems. Only one study, indeed, has so far addressed the ecological role of fungi in Antarctic marine habitats [85]. This knowledge gap highlights the importance to increase studies based on molecular and biochemical tools to better comprehend fungal diversity and ecology and to elucidate the nature and strength of the relationships between fungi and their hosts especially in extreme environments of the Earth, such as the Antarctic ecosystems.

## 3. Biotechnological Potential of Fungi Inhabiting Marine Antarctic Environments

The extreme conditions of Antarctic marine environments have forced microorganisms to evolve peculiar metabolic pathways as well as adaptive mechanisms, which allow them to thrive in cold ecosystems [140]. Psychrophilic and psychrotrophic fungi hold outstanding biological features arising from the harsh environmental conditions with which they must cope [141,142]. For these reasons, they are considered treasure of unique enzymes and bioactive molecules with an exceptional application potential [29,109,143,144,145,146]. Therefore, Antarctic fungi can greatly contribute to the discovery of new compounds of marine origin to be exploited in the industrial “white” and bio-pharmaceutical “red” biotechnology [28,147].

### 3.1. Antarctic Marine Fungi: Promising Candidates for Bioprospecting

With the increasing demand for novel antimicrobial and chemotherapeutic drugs, the discovery of biologically active molecules to improve human health is one of the most important challenges for mankind [148,149]. There is a general consensus that natural products offer extraordinary advantages over chemical molecules, and this makes marine microorganisms an astonishing potential source of new drugs [150,151,152,153,154]. Marine extremophilic microorganisms, including fungi, can also represent a huge reservoir of bioactive molecules that have recently triggered interest in bioprospecting research because of their promising therapeutical properties [21,22,29,109,152,155,156,157,158,159]. In this regard, marine fungi isolated from polar environments reported their ability to synthesize metabolites with unique structures and a wide range of biological activities, compared to mesophilic fungi, highlighting that psychrophilic fungi can be a new resource for several applications in biotechnology [28,30,160,161,162]. However, the search for natural bioactive products has been focused so far on a very small number of fungi isolated from Antarctic marine sediments, seawater and few organisms such as sponges and macroalgae [27].

Crude extracts of fungal strains have been isolated from fresh thalli of Antarctic macroalgal species and tested for their antibiotic, antifungal, antiviral, and antiparasitic activity [66]. Among these, extracts of two *Penicillium* species associated to the endemic macroalgae *Palmaria decipiens* and *Monostroma hariotii* contained compounds with high and selective antifungal and trypanocidal activities [68]. In addition, extracts of *Pseudogymnoascus* species, *Guehomyces pullulans,* and *Metschnikowia australis* showed high antifungal activity against *Candida albicans*, *Candida krusei*, and *Cladosporium sphaerospermum*, whereas the extract of *Penicillium steckii* greatly inhibited BHK-21 cell line expressing the yellow fever virus [68]. Moreover, *Geomyces* species associated with Antarctic marine sponges, have been suggested as a source of several promising antimicrobial and antitumoral compounds [82].

Interestingly, extracts of *Pseudogymnoascus* 5A-1C315IIII, isolated from marine sediments of Admiralty Bay (South Shetland Islands, Antarctica), can inhibit different phytopathogenic *Xanthomonas* species [57,58]. Another survey conducted on marine sediments collected at Deception Island (South Shetland Islands, Antarctica) led to the isolation of *Pseudogymnoascus* specie and *Simplicillium lamellicola,* which showed high and selective antifungal activity against *Paracoccidioides brasiliensis* [104].

Despite the considerable number of crude fungal extracts, only a few bioactive compounds have been actually tested so far (Table 1). Most of the research on the bioactive compounds carried out in Antarctic environments mainly focus on a few *Penicillium* strains, leaving most of the actual fungal biodiversity largely unexplored. The bioprospection of psychrophilic and psychrotolerant polar *Penicillium* strains have resulted in the collection of many promising bioactive molecules with a complex and peculiar structure and a broad range of biological activities, highlighting their outstanding potential [162]. For instance, eremophilane-type sesquiterpene isolated from *Penicillium* sp. PR19N-1, showed potent inhibitory activity against A549 tumor cells with IC50 value of 5.2 μM [51,163]. In a recent study, neuchromenin, extracted from *Penicillium glabrum* SF-7123, has shown great anti-inflammatory effects inactivating the NF-κB and p38 MAPK pathways in BV2 and RAW264.7 cells [50].

The fungus *Penicillium crustosum* HDN153086, isolated from Antarctic sediment from Prydz Bay, can synthesize a new diketopiperazine with moderate cytotoxic activity against K652 cell lines [61]. Interestingly, seven bioactive compounds extracted from *Penicillium citrinum* OUCMDZ4136, associated with the Antarctic krill *Euphausia superba,* showed moderate to strong cytotoxicity against A549, K562, and MCF-7 cell lines [83]. Conversely, *Pseudogymnoascus*, *Trichoderma, Aspergillus,* and other fungal genera isolated from macro-organisms living in Antarctic environments, have not received as much attention as the *Penicillium* genus. In fact, to date, only a few studies investigated the properties of bioactive molecules extracted from these isolated genera and tested their bioactivity [56,167,168,169]. For instance, different compounds have been extracted from *Pseudogymnoascus;* however, only two bioactive molecules (Geomycins B, C) from a sediment sample showed antifungal and antimicrobial activities [168].

### 3.2. Antarctic Fungi as Novel Source of Cold-Active Enzymes

Over the last 20 years, the interest in cold-adapted microorganisms as a source of new enzymes for industrial processes has intensely grown [170,171,172]. Nowadays, a considerable number of industrial processes and products take advantage of microbial enzymes [173,174]. Nevertheless, studies prospecting and characterizing enzymes have mainly focused on prokaryotes [175,176], while there is little information on enzymes from psychrophilic or psychrotolerant eukaryotes inhabiting Antarctic systems [162,177,178].

Despite the increasing demand for new biocatalysts, very few enzymes are currently isolated from extreme environments, the so-called “extremozymes” [179]. Cold extremozymes display a greater versatility and adaptability with respect to their non-extreme counterparts that can be advantageous for modern industries [180]. These enzymes are an optimal alternative to their mesophilic equivalents thanks to their higher stability under various physicochemical conditions (i.e., pH, temperature, salinity) and their advantage to reduce costs and energy consumption [181,182,183,184,185]. Indeed, the use of these enzymes represents an eco-friendly method compared to the chemical procedures employed in many industrial processes, allowing to avoid the use of organic solvents and other hazardous compounds that can be seriously harmful to the environment [186]. Nevertheless, only some of these potentially useful enzymes have been successfully introduced in the market so far, and their use is spreading rather slowly [187]. This is mainly due to several steps including chemical characterization, condition optimization, and process validation that need to be passed for commercializing novel biocatalysts [188]

Antarctic environments are a relatively new frontier for the isolation of cold-active enzymes. These molecules have distinct features that meet the need of green industry applications [162,177,189,190]. In this view, psychrophilic and psychrotolerant fungi are specialized in producing extracellular and intracellular cold active-enzymes [20,189,191], which they use to live in harsh conditions to degrade molecules and for the uptake of nutrients [141,170,192,193,194]. Recently, these cold-adapted enzymes have attracted growing interest because of their potential benefits in several industrial fields [175,185,195]. The main characteristics of fungal extremozymes are the high activity at low temperatures and thermolability, which have been manly gained the attention for being applied as detergent additives (e.g., lipases) for eco-friendly cold-water washing and for food, biofuel, and textile processing [184,185]. Among them, cold-adapted hydrolases (EC 3.x, proteases, lipases, cellulases, glycosidases) can be employed and useful in a variety of biotechnological processes (i.e., food, beverage, cleaning agents, textiles, biofuels, and pulp and paper; see Table 2). This class of enzymes is extremely important since it covers over 90% of the total industrial enzymes market [196,197].

*Tausonia pullulans* 17-1 isolated from Antarctic marine sediments can produce cold-active β-galactosidases, which can be a tool for hydrolyzing the lactose present in milk and milk derivatives at low temperatures in the milk-processing industry, allowing intolerant people to consume lactose-free foods and beverages [221,222].

Psychrophilic fungi isolated from Antarctic marine organisms are promising sources of cold-active xylanases, which have interesting applications in the food industry for bread-making as well as in agricultural industry and biofuel production [206,218,223,224,225]. For instance, the fungus *Cladosporium* sp. isolated from a marine sponge displayed high xylanase activity at a lower temperature than the mesophilic fungus *Penicillium purpurogenum* MYA-30, used as a control [218]. In addition, the *Penicillium* species isolated from different Antarctic marine organisms (i.e., sea stars, molluscs, macroalgae) were able to produce more than 10 U mL^−1^ of xylanase molecules after seven days of cultivation at 20.0 °C [206].

Microbial lipases are important enzymes employed in a variety of applications in the dairy, bakery, oil, meat and fish processing, and beverage industries, for enhancing the food quality, as well as for the detergent and cosmetic industry [226,227]. It is forecasted to reach a market size of 590.2 USD Million by 2023, with an annual growth rate of 6.8% from 2018 [228]. For example, Lipoclean^®^ marketed by Novozymes is a cold-active lipase that is suitable as a detergent additive for its activity at ≃20 °C, high stability in the presence of other enzymes, at alkaline pH and also resistance to oxidizing and chelating agents [229,230]. An interesting lipase activity was reported in some yeasts belonging to *Cryptococcus*, *Leucosporidium*, and *Metschnikowia* genera, which were isolated from Antarctic marine samples [47]. In particular, the highest activity (0.88 U mL^−1^) was observed in *Metschnikowia* sp. CRM 1589 isolated from marine sediments and *Salpa* sp. when cultured at 15 °C [47]. At the same temperature *Cryptococcus laurentii* L59, *Cryptococcus adeliensis* L121, and *Leucosporidium scottii* L117 showed an enzyme production between 0.1 and 0.23 U mL^−1^ after six days of incubation [54]. Generally, yeasts belonging to genera *Candida*, *Yarrowia*, and *Saccharomyces* can produce lipases [231]. In fact, *Candida antarctica* isolated from sediments of Lake Vanda in Antarctica (a lake permanently covered by ice) can produce two forms of distinct lipases (Lipase A and B), whose production has been patented in 2005 with different industrial and environmental applications [232,233,234].

Assays using Tween 80 as substrate for testing esterase activity testing identified *Cryptococcus,* *Metschnikowia, Rhodotorula*, *Leucosporidium*, and *Leucosporidiella* marine genera as cold-active esterase producers [45,208,235]. Hashim et al. (2018) discovered a new cold-active esterase-like protein with putative dienelactone hydrolase (GaDlh) activity produced by the psychrophilic yeast *Glaciozyma antarctica* isolated from sea ice near the Casey Research Station [205]. This pioneering study on the bioprospection of cold-active enzymes performing the isolation, heterologous expression, and biochemical characterization of recombinant GaDlh highlighted interesting cold-adapted features in the predicted protein structure at a temperature of 10 °C and pH 8.0 [205]. Overall, esterase enzymes are exploited in fine chemicals production and pharmaceutical industries for improving the production of optically pure compounds, such as ibuprofen, ketoprofen, and naproxen [236].

Since the beginning of the new millennium, very few studies have addressed the potential of Antarctic marine fungi as protease producers [237]. Proteases account for 60% of the total enzyme market and it is amongst the most precious commercial enzymes for the wide uses in different kind of industries (i.e., food, pharmacology, detergent) [237,238,239]. One of the first studies on microbial Antarctic proteases was carried out on *Leucosporidium antarcticum* 171, which can produce a novel extracellular serine protease, lap2 with an optimal temperature as low as 25 °C, high catalytic efficiency in the range 0–25 °C [214]. Afterward, halotolerant extracellular protease produced by *Rhodotorula mucilaginosa* L7 was characterized with optimal catalytic activity at 50 °C and pH 5.0, after a selection of protease positive strains isolated from marine organisms [54,212]. Recently, *Pseudogymnoascus* sp. CRM1533, isolated from Antarctic marine sediments, showed a protease activity of 6.21 U mL^−1^, even though further studies are needed to characterize the functional potential of this enzyme [47]. The genera *Metschnikowia*, *Cystofilobasidium,* and *Leucosporidiella,* associated with Antarctic marine sponges, displayed extracellular amylase between 4 and 20 °C, whereas only *Leucosporidiella* also showed protease activity [80].

Finally, different fungal genera such as *Penicillium*, *Cladosporium*, *Geomyces,* and *Pseudogymnoascus* isolated from Antarctic macroalgae and sponges showed carrageenolytic and agarolytic activities which can be useful in processes involving the extraction of the algal biomass for the production of bioethanol [69,210]. Among the tested strains, *Geomyces* sp. strain F09-T3-2 displayed also high activity pectinase: 121 U/mg after 5 days at 30 °C [210].

Overall, these findings indicate that the Antarctic marine ecosystems host promising fungal assemblages that display a wide array of unique and novel enzymes. These enzymes offer new horizons for a broad range of biotechnological applications and have great potential to reduce resource and energy consumption, thus promoting eco-sustainability. Obtaining further genetic and functional information on extremophilic fungi inhabiting Antarctic marine ecosystems, coupled with the development of specific bioinformatic pipelines for bioprospecting, are of fundamental importance for the identification of new fungal enzymes and molecules useful for enhancing the growth and competitiveness of the blue biotechnologies.

### 3.3. Emerging Bioprospecting Methods: Pitfalls and Future Perspectives

Isolation techniques usually employed for characterizing extremophilic fungi typically foster isolation of selected fungal taxa (e.g., faster-growing generalists, mesophilic strains), thus hampering our ability of bioprospection of natural molecules produced by the currently unculturable fungi [32,240,241]. Indeed, there is evidence that cultivability is a significant bottle-neck for the discovery of natural products from extremophilic marine fungi [242,243]. The implementation and development of methodologies aimed at the isolation of extremophilic fungi are urgently needed to fill this gap [244]. Novel cultivation methods as well as culture-independent approaches, can help to overcome current limitations in our understanding of the fungal biodiversity in extreme environments and in the discovery of new enzymes and molecules with biotechnological potential [245]. Indeed, culture-independent techniques coupled with genomics-based approaches are becoming valuable and fast tools to analyse the functional potential of fungal secondary metabolites useful for biotechnological applications [85,246,247]. Metagenomics, metatranscriptomics, and metaproteomics, as well as single-cell genomics, followed by heterologous expression of selected genes of potential interest, represent promising tools to shed new light on the possible biotechnological exploitation of still-uncultured Antarctic fungi [248,249,250,251]. Finally, the bioinformatics mining of the still poorly described but rich genetic biodiversity of Antarctic fungi will certainly enhance the rate of discovery of bioactive molecules potentially useful for biotechnological purposes [30,252].

## Figures and Tables

**Table 1:** Bioactive molecules isolated from Antarctic marine fungi. Available chemical structures of the bioactive compounds have been downloaded from [164] and used to produce Figures S1–S3 for ease of visualization.

Fungal Taxa	Product	Bioactivity	Source	Ref.
*Penicillium citrinum* OUCMDZ4136	2,4-Dihydroxy-3,5,6-trimethylbenzoic acid; Citreorosein; Pinselin; Citrinin; Dihydrocitrinone; Pennicitrinone A; Quinolactacin A1	Cytotoxic activities against MCF-7, A549, K562 cell lines	Antarctic krill *Euphasia superba*	[83]
*Penicillium citreonigrum* SP-6	Diketopiperazine, phenols	Inhibitory activity against HCT116 cancer cell line	Marine sediment, Great Wall Station	[165]
*Penicillium crustosum* HDN153086	Diketopiperazine	Cytotoxic activities against K562 cell line	Marine sediment, Pridz Bay	[61]
*Penicillium crustosum* PRB-2	Penilactone A	NF-KB inhibitory activities of HCT-8, Bel-7402, BGC-823, A549 and A2780 tumor cell lines	Deep-sea sediment, Prydz Bay	[46]
*Penicillium glabrum* SF-7123	Citromycetin derivative, neuchromenin; myxotrichin C, deoxyfunicone;	Anti-inflammatory; tyrosine phosphatase 1B inhibition	Marine sediment, Ross Sea	[50]
*Penicillium granulatum* MCCC 3A00475	Spirograterpene A	Antiallergic effect on immunoglobulin E (IgE)-mediated rat mast RBL-2H3 cells	Deep-sea sediment, Prydz Bay	[52]
*Penicillium* sp. PR19N-1	Chlorinated eremophilane sesquiterpenes, eremofortine C, eremophilane-type sesquiterpenes, eremophilane-type lactam	Cytotoxic activity against HL-60 and A549 cancer cell lines	Deep-sea sediment, Prydz Bay	[51,163]
*Penicillium* sp. S-1–18	Butanolide A, guignarderemophilane F, xylarenone A	Butanolide: inhibitory activity against tyrosine phosphatase 1B; xylarenone A: antitumor activity against HeLa and HepG2 cells and growth-inhibitory effects against pathogenic microbes	Sea-bed sediment	[60,166]
*Penicillium* sp. UFMGCB 6034 and UFMGCB 6120	Aromatic compounds	Antifungal and trypanocidal activities	Macroalgae: *Palmaria decipiens* and *Monostroma hariotii*	[66]
*Pseudogymnoascus* sp.	Pseudogymnoascin A, B, C, 3-nitroasterric acid; Geomycins B, C	Antibacterial and antifungal activities	Sponge genus *Hymeniacidon*	[167,168]
*Trichoderma asperellum*	Asperelines A-F, peptaibols	Not assayed	Marine sediment, Penguin Island	[56,169]

**Table 2:** Examples of extremophilic fungi as a source of cold-adapted enzymes utilized in industrial applications. The fungal taxa reported are isolated from Antarctic marine environments: seawater, marine sediments, and organisms.

Enzyme	Reaction	Fungi	Source of (Isolate) Sample	Applications/Potential Uses	Ref.
Carragenase (EC 3.2.1.83)	Hydrolysis of 1,4-β-linkages between galactose 4-sulfate and 3,6-anhydro-galactose to produce kappa-carrageenans	*Pseudogymnoascus* sp. UFMGCB 10054	Macroalga: *Iridaea cordata*,	Biomedical field, textile industry, bioethanol production, and detergent additive	[69]
Cellulase (EC 3.2.1.4)	Cellulose hydrolysis into glucose	*Cystofilobasidium infirmominiatum* 071209-E8-C1-liblev; *Metschnikowia australis*, *Rhodotorula glacialis*; *Candida spencermartinsiae*, *Leucosporidiella creatinivora*, *Leucosporidium scottii*	Marine sponge: *Tedania*; marine sediments; seawater	Food industry, animal feed, beer and wine, textile and laundry, pulp and paper industry, agriculture, biofuel, pharmaceutical industries, and waste management	[45,80]
Chitinase (EC 3.2.1.14)	Cleavage of glycosidic linkages in chitin and chitodextrins generating chitooligosaccharides	*Lecanicillium muscarium* CCFEE-5003; *Glaciozyma antarctica* PI12	Shrimp wastes; seawater	Cosmetic, pharmaceutic fields, fermentation research, and biomedicine	[45,198,199,200,201,202]
Endo-β-1,3(4)-glucanase (EC 3.2.1.6)	Endohydrolysis of (1→3)- or (1→4)-linkages in β-D-glucans	*Glaciozyma antarctica* PI12	Seawater	Brewing and animal, feed-stuff industry, biofuel production, and pharmaceuticals	[202,203,204]
Esterase (EC 3.1.1.1)	Hydrolyis of short acyl-chain soluble esters	*Cryptococcus victoriae*, *Metschnikowia australis*, *Rhodotorula glacialis*, *Leucosporidium scottii*, *Leucosporidiella creatinivora*; *Glaciozyma antarctica*	Marine sediments; seawater, sea ice	Paper bleaching, bioremediation, degradation, and removal of xenobiotics and toxic compounds	[45,205]
Invertase (EC 3.2.1.26)	Hydrolysis of the terminal non-reducing β-fructofuranoside residue in sucrose, raffinose and related β-D-fructofuranosides	*Glaciozyma antarctica* 17 (formerly *Leucosporidium antarcticum*)	Seawater	Beverage, confectionary, bakery, invert sugar, high fructose syrup, artificial honey, calf feed, food for honeybees	[38]
Laccase (EC 1.10.3.2)	Oxidation of phenolic compound like lignin	*Cadophora malorum* A2B, *Cadophora malorum* AS2A, *Cadophora luteo*-*olivacea* P1	Marine sediments	Biosensors, microfuel and bioelectrocatalysis, food, pharmaceutic, cosmetic, pulp and paper, textile industries, and bioremediation	[206]
Lignin peroxidase (EC 1.11.1.14)	Oxidative breakdown of lignin	*Cadophora malorum* M7, *Cadophora* sp. OB-4B	Marine sediments	Pulp and paper, cosmetics (treatment of hyperpigmentation, and skin-lightening through melanin oxidation), textile, bioremediation (degradation of azo, heterocyclic, reactive, and polymeric dyes, xenobiotic, and pesticides), and bioethanol production	[206]
Lipase (EC 3.1.1.3)	Hydrolysis of long-chain triacylglycerol substances with the formation of an alcohol and a carboxylic acid	*Leucosporidium scottii* L117, *Metschnikowia* sp. CRM1589; *Mrakia blollopis* SK-4; *Cystofilobasidium infirmominiatum* 071209-E8-C1-IIa-lev and isolate 131209-E2A-C1-II-lev; *Metschnikowia australis* 131209-E3-C1-(GPY)-lev and isolate 131209-E2A-C4-II-lev; *Rhodotorula pinicola* 071209-E4-C9-lev; *Candida zeylanoides*, *Cryptococcus victoriae*, *Leucosporidiella creatinivora*, *Leucosporidium scottii*, *Candida sake*, *Candida spencermartinsiae*	Marine sediments; Algal mat in sediment; marine sponges: *Tedania*, *Hymeniacidon*, *Dendrilla*; Seawater	Food, beverage, detergent, biofuel production, animal feed, textiles, leather, paper processing, and cosmetic industry	[45,47,48,207,208,209]
L-asparaginase (EC 3.5.1.1)	Degradation of asparagine into ammonia and aspartate	*Cosmospora* sp 0B4B, *Cosmospora* sp 0B1B, *Cosmospora* sp 0B2, *Geomyces* sp. S2B	Marine sediments	Food industry and medical applications as anti-cancer, antimicrobial, infectious diseases, autoimmune diseases	[206]
Pectinase (EC 3.2.1.15)	Hydrolisis of polysaccharides to produce pectate and other galacturonans	*Geomyces* sp. strain F09-T3-2, *Pseudogymnoascus* sp., *Cladosporium* sp. *F09-T12-1, Cryptococcus victoriae*, *Leucosporidiella muscorum*, *Metschnikowia australis*, *Rhodotorula glacialis*; *Leucosporidiella creatinivora*, *Leucosporidium scottii*	Marine sponges; marine sediments; Seawater	Food and textile industry, coffee and tea fermentation, wine processing, oil extraction, vegetable and fruit processing industry for juice clarification, color, and yield enhancer. Applications in paper and pulp making, recycling of wastepaper, pretreatment of pectic wastewaters, and retting of plant fibers	[45,82,210]
Phytase (EC 3.1.3.26)	Hydrolysis of phytate to produce phosphorylated myo-inositol derivatives	*Rhodotorula mucilaginosa* JMUY14	Deep-sea sediments	Food and feed industry, pharmaceutical use as neuro protective agents, anti-inflammatory, antioxidant and anti-cancer agents	[211]
Protease (EC 3.4)	Cleavage of peptide bonds	*Rhodotorula mucilaginosa* L7; *Pseudogymnoascus* sp. CRM1533, *Leucosporidiella muscorum*; *Leucosporidiella* sp. 131209-E2A-C3-II-lev, *Leucosporidiella creatinivora* 071209-E8-C4-II-lev; *Rhodotorula glacialis*; *Leucosporidiella creatinivora*, *Leucosporidium scottii*	Marine macroalgae; marine sediments; marine sponges: *Tedania*, *Hymeniacidon*; Seawater	Food, feed, pharmacology (anticancer and antihemolytic activity) cosmetic (keratin-based preparation) industries, cleaning processes (e. g. detergent additive), waste management	[45,47,48,212,213]
Protease (Subtilase) (EC 3.4.21)	Cleavage of peptide bonds	*Glaciozyma antarctica* 17 (formerly *Leucosporidium antarcticum*)	Sub-glacial waters (depth of 200 m)	Food and beverage industries	[214,215]
Transglutami-nase (EC 2.3.2.13)	Acyl transfer reaction between gamma-carboxyamide groups of glutamine residues in proteins and various primary amines	*Penicillium chrysogenum*	Marine macroalga *Gigartinas kosttbergii*	Food, pharmaceutical, leather, textile, biotechnology industry, biomedical research	[216]
Xylanase (EC 3.2.1.8)	Hydrolysis of the main chain of xylan to oligosaccharides, which in turn are degraded to xylose	*Cladosporium* sp.; *Penicillium* sp. E2B *Penicillium* sp. N5, *Penicillium* sp. E2-1	Marine sponge; marine sediments	Food (bread making), feed, paper and pulp industries, and also used to increase the sugar recovery from agricultural residues for biofuel production	[206,217,218]
α-amylase (EC 3.2.1.1)	Cleavage of α-1,4-glycosidic linkages within starch molecules, which generate smaller polymers of glucose units	*Glaciozyma antarctica* PII12 (formerly *Leucosporidium antarcticum*); *Cystofilobasidium infirmominiatum* 071209-E8-C1-IIa-lev, 131209-E2A-C1-II-lev, 131209-E2A-C5-II-lev and isolate 071209-E8-C1-IIb-lev; *Metschnikowia* australis 071209-E8-C3-II-lev and isolate 071209-E8-C1-II-lev; *Leucosporidiella* sp. 131209-E2A-C3-II-lev	Seawater; marine sponges: *Tedania*, *Hymeniacidon*	Pharmaceutical and chemical industry; employed as additives in processed food, in detergents for cold washing, in waste-water treatment, in bioremediation in cold climates, and in molecular biology protocols	[80,202,219]
β-agarase (EC 3.2.1.81)	Hydrolysis of beta-(1–>4) linkages of agarose to produce oligosaccharides	*Penicillium* sp., *Cladosporium* sp. 2, *Penicillium* sp., *Pseudogymnoascus* sp. UFMGCB 10054, *Doratomyces* sp.	Macroalgae: *Ascoseira mirabilis*, *Georgiella confluens*, *Iridaea cordata*, *Palmaria decipiens*	Food, cosmetic, medical industries, and as a tool enzyme for biological, physiological, and cytological studies	[69]
β-galactosidase (EC 3.2.1.23)	Hydrolysis of lactose into its constituent monosaccharides	*Tausonia pullulans* 17-1 (formerly *Guehomyces pullulans*)	Marine sediments	Food, biofuel, and agricultural industries; surfactant production	[220]

## Supplementary Materials

The following are available online at https://www.mdpi.com/article/10.3390/jof7050391/s1, Figures S1–S3: Chemical structures of available bioactive compounds; Table S1: Coordinates of different Antarctic samples collected for fungal identification; Table S2: Fungal taxa isolated with culture-dependent methods and identified through metagenomic analyses.
