# Supplementary material for: Diversity, Ecological Role and Biotechnological Potential of Antarctic Marine Fungi
Source: J Fungi (Basel). 2021 May 17;7(5):391. doi: 10.3390/jof7050391 (PMC8157204; doi:10.3390/jof7050391)

Figure S1

2,4-Dihydroxy-3,5,6-trimethylbenzoic acid

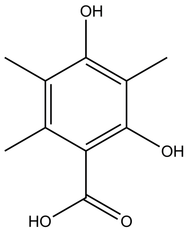

Citreorosein

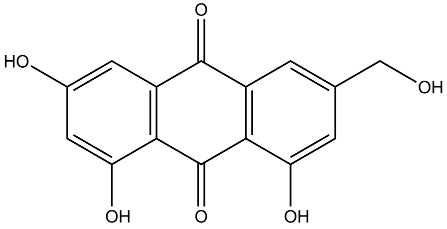

Pinselin

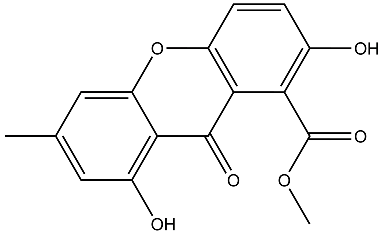

Citrinin

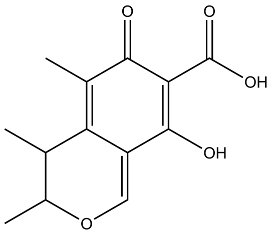

Dihydrocitrinone

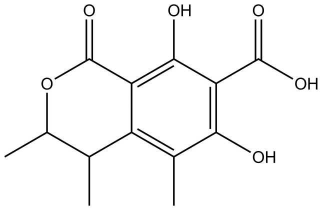

Pennicitrinone A

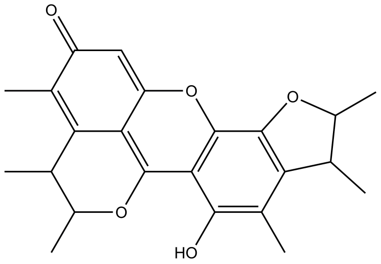

Penilactone A

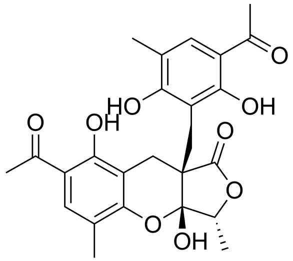

Diketopiperazine

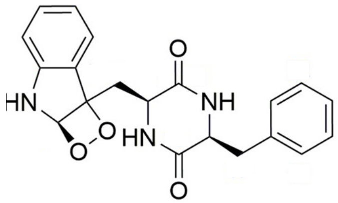

Phenolic compound

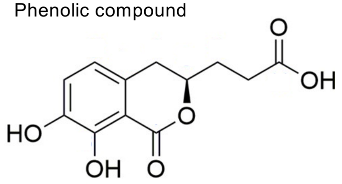

Neuchromenin

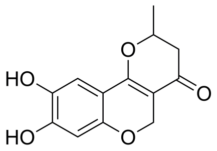

Deoxyfunicone

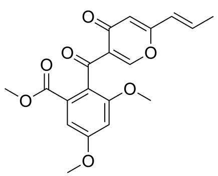

Spirograterpene A

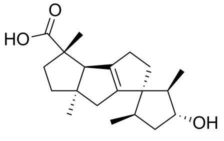

Butanolide

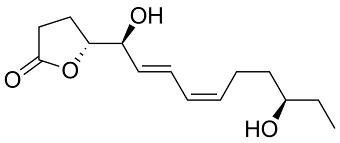

Guignarderemophilane F

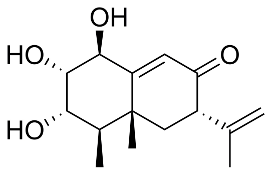

Xylarenone A

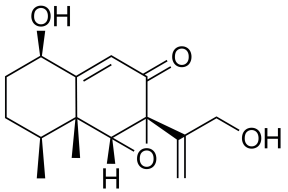

Pseudogymnoascin A

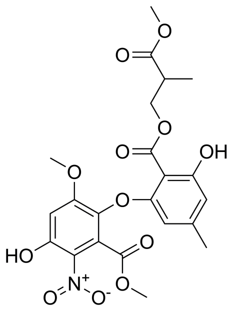

Supplement: Supplementary file 1 [file jof-07-00391-s001.zip › Figure S1.pdf]
