# Supplementary material for: Diversity, Ecological Role and Biotechnological Potential of Antarctic Marine Fungi
Source: J Fungi (Basel). 2021 May 17;7(5):391. doi: 10.3390/jof7050391 (PMC8157204; doi:10.3390/jof7050391)
Supplement: Supplementary file 1 [file jof-07-00391-s001.zip › FIgure S2.pdf]

Figure S2

Pseudogymnoascin B

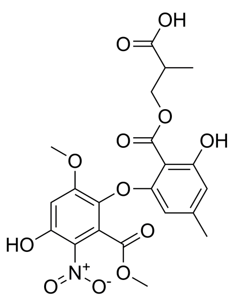

Pseudogymnoascin C

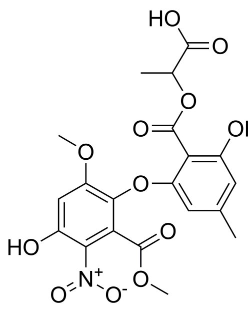

3-Nitroasterric acid

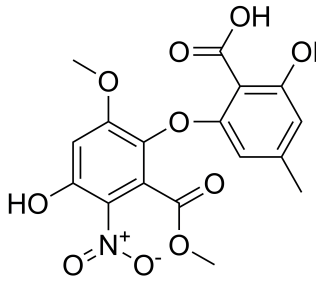

Geomycin B

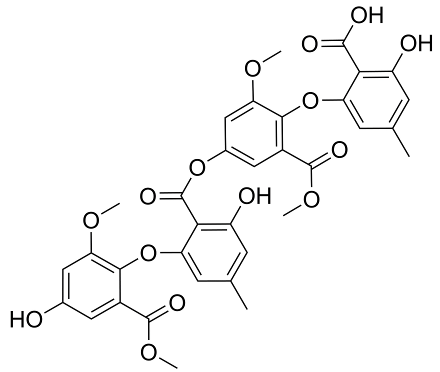

Geomycin C

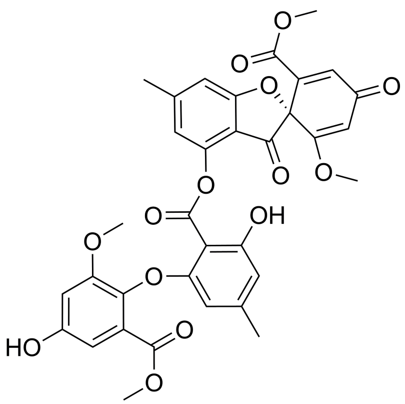

Aspereline A

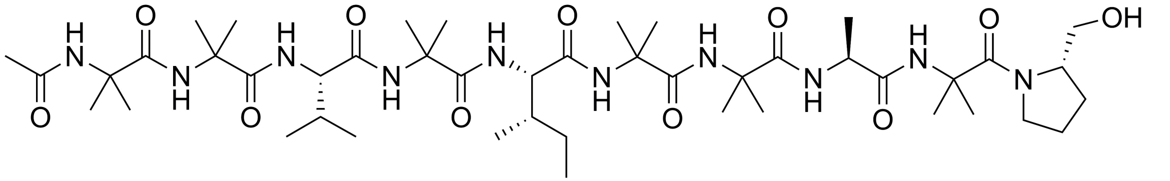

Aspereline B

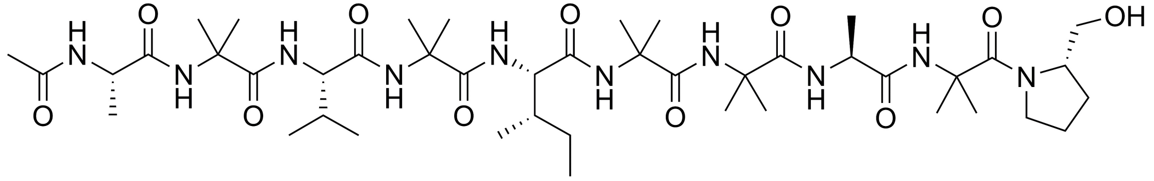

Citromycetin derivative

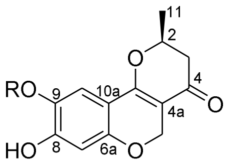

R = CH_3
