# Supplementary figures and images for: Diversity, Ecological Role and Biotechnological Potential of Antarctic Marine Fungi
Source: J Fungi (Basel). 2021 May 17;7(5):391. doi: 10.3390/jof7050391 (PMC8157204; doi:10.3390/jof7050391)

Figure S3

Aspereline C

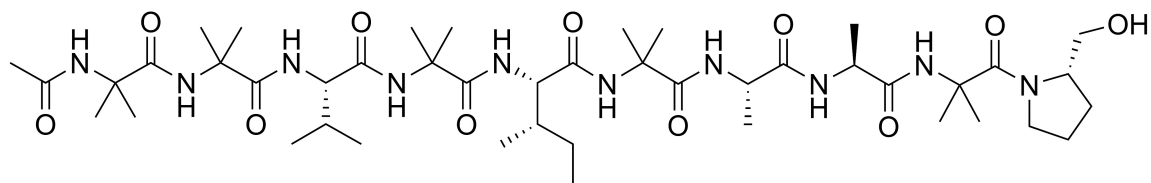

Aspereline D

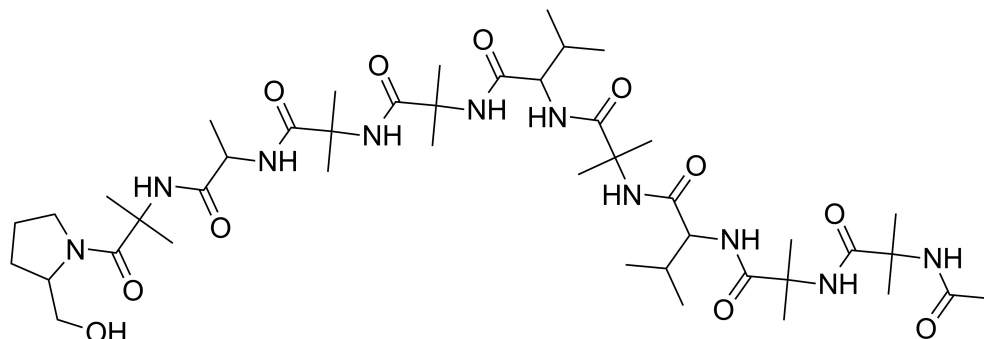

Aspereline E

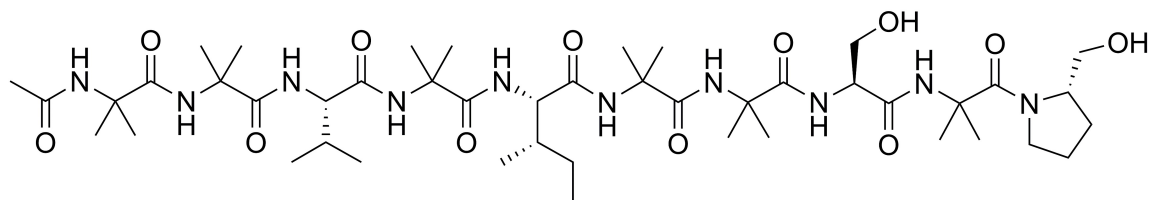

Aspereline F

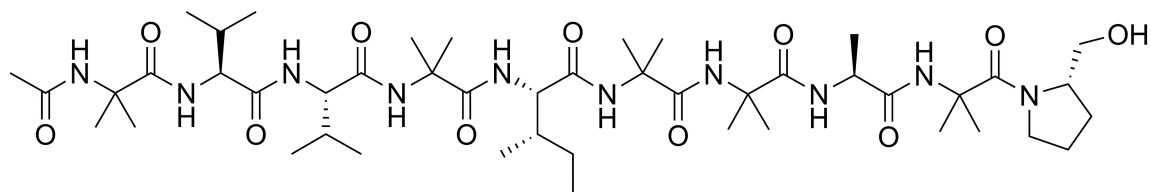

Supplement: Supplementary file 1 [file jof-07-00391-s001.zip › Figure S3.pdf]
